# 2-(4-Chloro­phen­yl)-6-meth­oxy­chroman-4-one

**DOI:** 10.1107/S1600536810035816

**Published:** 2010-09-11

**Authors:** Jerry P. Jasinski, Albert E. Pek, B. Narayana, H. S. Yathirajan, Prakash S. Nayak

**Affiliations:** a Department of Chemistry, Keene State College, 229 Main Street, Keene, NH 03435-2001, USA; b Department of Studies in Chemistry, Mangalore University, Mangalagangotri, Mysore 570 006 India; c Department of Studies in Chemistry, University of Mysore, Manasagangotri, Mysore 570 006, India

## Abstract

In the title mol­ecule, C_16_H_13_Cl O_3_, the two aromatic rings form a dihedral angle of 65.3 (1)°. In the crystal structure, weak inter­molecular C—H⋯O hydrogen bonds link the mol­ecules into centrosymmetric dimers, which are further packed into columns propagating in [100] by weak C—H⋯π inter­actions.

## Related literature

For the pharmacological and alkyl­ating properties of chromenes (benzopyrans) and their derivatives and for their use as synthons for the synthesis of natural products, see: Brooks (1998 ▶); Chenera *et al.* (1993 ▶); Ellis *et al.* (1997 ▶); Gabor *et al.* (1988 ▶); Hatakeyama *et al.* (1988 ▶); Hyana & Saimoto, *et al.* (1987 ▶); Kooijman *et al.* (1984 ▶); Liu *et al.* (2007 ▶); Tang *et al.* (2007 ▶); Valenti *et al.* (1993 ▶). For related structures, see: Brito *et al.* (2008 ▶); Butcher *et al.* (2007 ▶); Li *et al.* (2007 ▶); Nallasivam *et al.* (2009 ▶); Hao *et al.* (2010 ▶). For bond-length data, see: Allen *et al.* (1987 ▶).
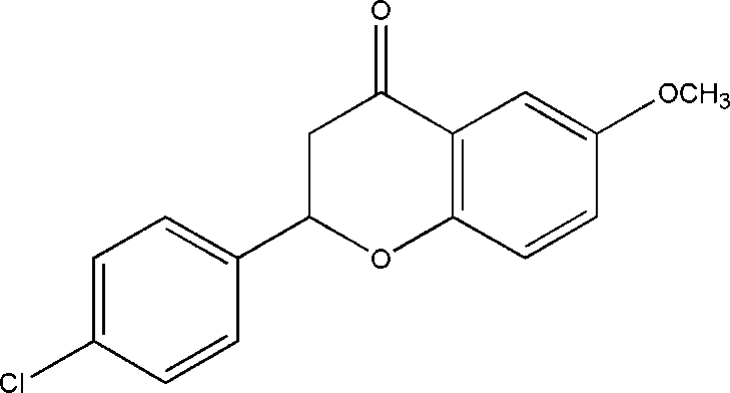


## Experimental

### 

#### Crystal data

C_16_H_13_ClO_3_
*M* *_r_* = 288.71
Triclinic, 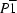

*a* = 5.0188 (3) Å
*b* = 12.0138 (7) Å
*c* = 12.3708 (7) Å
α = 108.035 (5)°
β = 98.379 (4)°
γ = 91.820 (5)°
*V* = 699.33 (7) Å^3^
*Z* = 2
Cu *K*α radiation
μ = 2.46 mm^−1^
*T* = 293 K
0.40 × 0.35 × 0.20 mm

#### Data collection

Oxford Diffraction Xcalibur diffractometer with a Ruby (Gemini Cu) detector
Absorption correction: multi-scan (*CrysAlis RED*; Oxford Diffraction, 2007 ▶) *T*_min_ = 0.590, *T*_max_ = 1.000
4431 measured reflections
2733 independent reflections
2318 reflections with *I* > 2σ(*I*)
*R*_int_ = 0.018

#### Refinement

*R*[*F* ^2^ > 2σ(*F* ^2^)] = 0.049
*wR*(*F* ^2^) = 0.156
*S* = 1.62
2733 reflections
183 parameters
H-atom parameters constrained
Δρ_max_ = 0.17 e Å^−3^
Δρ_min_ = −0.39 e Å^−3^

### 

Data collection: *CrysAlis PRO* (Oxford Diffraction, 2007 ▶); cell refinement: *CrysAlis RED* (Oxford Diffraction, 2007 ▶); data reduction: *CrysAlis RED*; program(s) used to solve structure: *SHELXS97* (Sheldrick, 2008 ▶); program(s) used to refine structure: *SHELXL97* (Sheldrick, 2008 ▶); molecular graphics: *SHELXTL* (Sheldrick, 2008 ▶); software used to prepare material for publication: *PLATON* (Spek, 2009 ▶).

## Supplementary Material

Crystal structure: contains datablocks global, I. DOI: 10.1107/S1600536810035816/cv2754sup1.cif

Structure factors: contains datablocks I. DOI: 10.1107/S1600536810035816/cv2754Isup2.hkl

Additional supplementary materials:  crystallographic information; 3D view; checkCIF report

## Figures and Tables

**Table 1:** Hydrogen-bond geometry (Å, °) *Cg*1 is the centroid of the C7–C12 ring.

*D*—H⋯*A*	*D*—H	H⋯*A*	*D*⋯*A*	*D*—H⋯*A*
C7—H7⋯O2^i^	0.98	2.50	3.260 (2)	135
C8—H8*B*⋯*Cg*1^ii^	0.97	2.69	3.5709 (18)	151

Symmetry codes: (i) 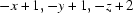; (ii) 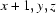.

**Fig. 1.**
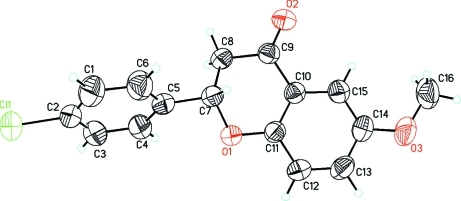
Molecular structure of (I) showing the atom labeling scheme and 50% probability displacement ellipsoids.

**Fig. 2.**
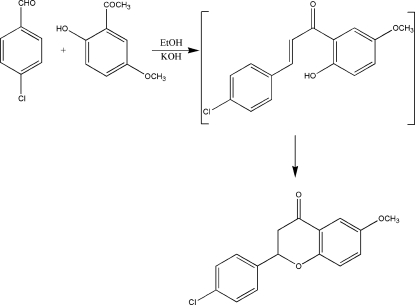
Reaction scheme for (I).

## supplementary crystallographic information

### Comment

Chromenes (benzopyrans) and their derivatives exhibit a wide spectrum of biological and pharmacological properties including spasmolytic, antisterility, anti-arrhytmic, cardionthonic, antiviral, anticancer and alkylating properties (Gabor *et al.*, 1988; Brooks, 1998; Valenti *et al.*, 1993; Hyana & Saimoto, *et al.* 1987; Tang *et al.*, 2007). In addition, polyfunctionalized chromene units are present in numerous natural products (Hatakeyama *et al.*, 1988). Chromanone derivatives are important synthons for the synthesis of natural products such as brazillin, hematoxylin, ripariochromene and clausenin (Kooijman *et al.*, 1984; Ellis *et al.*, 1997; Chenera *et al.*, 1993; Liu *et al.*, 2007). The crystal structures of some rleated chromene derivatives *viz*., 7-hydroxy-4-methyl-2*H*-chromen-2-one monohydrate (Butcher *et al.*, 2007), 5,7-dimethoxy-3-(4-methoxyphenyl)-4*H*-chromen-4-one (Li *et al.*, 2007), 5,7-dimethoxy-2-phenyl-4*H*-chromen-4-one (Nallasivam *et al.*, 2009), 5-hydroxy-7-methoxy-4*H*-chromen-4-one (Brito *et al.*, 2008) and 3-methyl-4*H*-chromen-4-one (Hao *et al.*, 2010) have been reported. In view of the importance of chromene derivatives, the crystal structure of the title compound, (I), is reported.

In (I), 4-chloro phenyl ring is found bonded to a 6-methoxy-2,3-dihydro-4*H*-chromen-4-one ring at C7 which is in an S configuration (Fig.1). The fused pyran ring in the benzopyran moiety adopts a slightly distorted envelope conformation with puckering parameters Q, θ and φ of 0.4973 (16)A%, 122.19 (19)°, and 243.0 (2)°, respectively. The dihedral angles between the mean planes of the benzene and benzopyran rings is 65.3 (1)°. Bond distances (Allen *et al.*, 1987) and angles are in normal ranges.  Weak C—H···O hydrogen bond and C—H···π intermolecular interactions (where *Cg*1 is the centroid of ring C7—C12) are observed which contribute to crystal packing (Table 1).

### Experimental

To a mixture of 1-(2-hydroxy-5-methoxyphenyl)ethanone (1.66 g, 0.01 mol) and *p*-chloro benzaldehyde (1.4 g, 0.01 mol) in 30 ml e thanol, 10 ml of 10% potassium hydroxide solution was added and stirred at 5–10 C° for 24 h (Fig. 2). The precipitate formed was collected by filtration and purified by recrystallization from ethanol. Single crystals were grown from DMF by the slow evaporation method and the yield of the compound was 75%. (m.p. 378 K).

### Refinement

All of the H atoms were placed in their calculated positions and then refined using the riding model with C—H = 0.93–0.97 Å, and with *U*_iso_(H) = 1.19–1.50*U*_eq_(C).

### Crystal data

**Table:**

C_16_H_13_ClO_3_	*Z* = 2
*M**_r_* = 288.71	*F*(000) = 300
Triclinic, *P*1	*D*_x_ = 1.371 Mg m^−^^3^
Hall symbol: -P 1	Cu *K*α radiation, λ = 1.54178 Å
*a* = 5.0188 (3) Å	Cell parameters from 2808 reflections
*b* = 12.0138 (7) Å	θ = 4.5–74.2°
*c* = 12.3708 (7) Å	µ = 2.46 mm^−^^1^
α = 108.035 (5)°	*T* = 293 K
β = 98.379 (4)°	Block, colourless
γ = 91.820 (5)°	0.40 × 0.35 × 0.20 mm
*V* = 699.33 (7) Å^3^	

### Data collection

**Table:**

Oxford Diffraction Xcalibur with a Ruby (Gemini Cu) detector diffractometer	2733 independent reflections
Radiation source: Enhance (Cu) X-ray Source	2318 reflections with *I* > 2σ(*I*)
graphite	*R*_int_ = 0.018
Detector resolution: 10.5081 pixels mm^-1^	θ_max_ = 74.3°, θ_min_ = 4.5°
ω scans	*h* = −6→5
Absorption correction: multi-scan (*CrysAlis RED*; Oxford Diffraction, 2007)	*k* = −14→14
*T*_min_ = 0.590, *T*_max_ = 1.000	*l* = −13→15
4431 measured reflections	

### Refinement

**Table:**

Refinement on *F*^2^	Primary atom site location: structure-invariant direct methods
Least-squares matrix: full	Secondary atom site location: difference Fourier map
*R*[*F*^2^ > 2σ(*F*^2^)] = 0.049	Hydrogen site location: inferred from neighbouring sites
*wR*(*F*^2^) = 0.156	H-atom parameters constrained
*S* = 1.62	*w* = 1/[σ^2^(*F*_o_^2^) + (0.072*P*)^2^] where *P* = (*F*_o_^2^ + 2*F*_c_^2^)/3
2733 reflections	(Δ/σ)_max_ < 0.001
183 parameters	Δρ_max_ = 0.17 e Å^−^^3^
0 restraints	Δρ_min_ = −0.39 e Å^−^^3^

### Special details

**Table:**

Geometry. All e.s.d.'s (except the e.s.d. in the dihedral angle between two l.s. planes) are estimated using the full covariance matrix. The cell e.s.d.'s are taken into account individually in the estimation of e.s.d.'s in distances, angles and torsion angles; correlations between e.s.d.'s in cell parameters are only used when they are defined by crystal symmetry. An approximate (isotropic) treatment of cell e.s.d.'s is used for estimating e.s.d.'s involving l.s. planes.
Refinement. Refinement of *F*^2^ against ALL reflections. The weighted *R*-factor *wR* and goodness of fit *S* are based on *F*^2^, conventional *R*-factors *R* are based on *F*, with *F* set to zero for negative *F*^2^. The threshold expression of *F*^2^ > σ(*F*^2^) is used only for calculating *R*-factors(gt) *etc*. and is not relevant to the choice of reflections for refinement. *R*-factors based on *F*^2^ are statistically about twice as large as those based on *F*, and *R*- factors based on ALL data will be even larger.

### Fractional atomic coordinates and isotropic or equivalent isotropic displacement parameters (Å^2^)

**Table:**

	*x*	*y*	*z*	*U*_iso_*/*U*_eq_	
Cl1	1.44159 (18)	1.09772 (6)	1.41015 (7)	0.1128 (4)	
C15	0.1890 (4)	0.52826 (16)	0.74092 (15)	0.0510 (4)	
H14	0.1871	0.4469	0.7205	0.061*	
C10	0.3876 (3)	0.59907 (15)	0.82937 (14)	0.0445 (4)	
C11	0.3896 (3)	0.72078 (15)	0.85979 (14)	0.0457 (4)	
C5	0.8624 (4)	0.83325 (15)	1.12894 (15)	0.0486 (4)	
C4	1.0438 (4)	0.90581 (17)	1.10279 (18)	0.0588 (5)	
H4	1.0451	0.9004	1.0262	0.071*	
C3	1.2237 (5)	0.98652 (19)	1.1888 (2)	0.0698 (6)	
H3	1.3442	1.0356	1.1706	0.084*	
C2	1.2221 (5)	0.99320 (18)	1.3011 (2)	0.0703 (6)	
C6	0.8664 (5)	0.8411 (2)	1.24293 (19)	0.0714 (6)	
H6	0.7459	0.7924	1.2617	0.098 (9)*	
C1	1.0475 (6)	0.9205 (2)	1.3295 (2)	0.0830 (7)	
H1	1.0510	0.9247	1.4061	0.100*	
C12	0.1994 (4)	0.77156 (17)	0.79994 (16)	0.0547 (4)	
H11	0.2034	0.8528	0.8183	0.066*	
C14	−0.0029 (4)	0.57890 (18)	0.68435 (16)	0.0564 (5)	
C13	0.0067 (4)	0.70101 (19)	0.71392 (17)	0.0610 (5)	
H12	−0.1204	0.7352	0.6744	0.073*	
C7	0.6684 (3)	0.74364 (14)	1.03660 (15)	0.0454 (4)	
H7	0.5149	0.7247	1.0703	0.055*	
C9	0.5995 (3)	0.54557 (14)	0.88808 (15)	0.0453 (4)	
C8	0.7957 (3)	0.63138 (15)	0.98271 (15)	0.0483 (4)	
H8B	0.8568	0.5958	1.0415	0.058*	
H8A	0.9521	0.6497	0.9517	0.058*	
O1	0.5707 (2)	0.79532 (10)	0.94837 (11)	0.0500 (3)	
O2	0.6175 (3)	0.43987 (11)	0.86011 (12)	0.0597 (4)	
O3	−0.2094 (3)	0.51981 (15)	0.59887 (13)	0.0759 (5)	
C16	−0.2392 (5)	0.3957 (2)	0.5693 (2)	0.0841 (7)	
H15A	−0.0793	0.3635	0.5425	0.126*	
H15B	−0.3924	0.3652	0.5094	0.126*	
H15C	−0.2661	0.3745	0.6358	0.126*	

### Atomic displacement parameters (Å^2^)

**Table:**

	*U*^11^	*U*^22^	*U*^33^	*U*^12^	*U*^13^	*U*^23^
Cl1	0.1341 (7)	0.0652 (4)	0.1049 (5)	−0.0140 (4)	−0.0625 (5)	0.0169 (3)
C15	0.0503 (10)	0.0501 (9)	0.0500 (9)	0.0020 (7)	0.0083 (8)	0.0124 (8)
C10	0.0406 (8)	0.0485 (9)	0.0462 (9)	0.0040 (7)	0.0085 (7)	0.0170 (7)
C11	0.0419 (8)	0.0491 (9)	0.0480 (9)	0.0028 (7)	0.0056 (7)	0.0192 (7)
C5	0.0470 (9)	0.0455 (9)	0.0520 (9)	0.0090 (7)	0.0023 (7)	0.0157 (7)
C4	0.0606 (11)	0.0546 (11)	0.0586 (11)	−0.0020 (9)	0.0036 (9)	0.0181 (9)
C3	0.0647 (13)	0.0559 (11)	0.0834 (15)	−0.0077 (9)	−0.0067 (11)	0.0239 (10)
C2	0.0761 (14)	0.0470 (10)	0.0716 (13)	0.0032 (9)	−0.0259 (11)	0.0129 (9)
C6	0.0796 (15)	0.0738 (14)	0.0572 (12)	−0.0087 (11)	0.0004 (10)	0.0222 (10)
C1	0.1029 (19)	0.0826 (16)	0.0531 (12)	−0.0017 (14)	−0.0109 (12)	0.0182 (11)
C12	0.0571 (11)	0.0532 (10)	0.0569 (10)	0.0092 (8)	0.0031 (8)	0.0242 (8)
C14	0.0500 (10)	0.0679 (12)	0.0459 (9)	0.0011 (8)	0.0007 (7)	0.0139 (8)
C13	0.0573 (11)	0.0707 (12)	0.0551 (10)	0.0127 (9)	−0.0032 (9)	0.0251 (9)
C7	0.0432 (9)	0.0458 (9)	0.0496 (9)	0.0051 (7)	0.0050 (7)	0.0194 (7)
C9	0.0425 (9)	0.0446 (9)	0.0515 (9)	0.0056 (7)	0.0108 (7)	0.0175 (7)
C8	0.0414 (9)	0.0490 (9)	0.0555 (10)	0.0071 (7)	0.0040 (7)	0.0195 (8)
O1	0.0508 (7)	0.0434 (6)	0.0553 (7)	0.0009 (5)	−0.0033 (5)	0.0206 (5)
O2	0.0647 (8)	0.0437 (7)	0.0688 (8)	0.0083 (6)	0.0041 (6)	0.0182 (6)
O3	0.0662 (9)	0.0812 (11)	0.0629 (9)	−0.0018 (7)	−0.0170 (7)	0.0113 (8)
C16	0.0807 (16)	0.0814 (16)	0.0677 (14)	−0.0132 (13)	−0.0115 (12)	0.0041 (12)

### Geometric parameters (Å, °)

**Table:**

Cl1—C2	1.744 (2)	C1—H1	0.9300
C15—C14	1.376 (3)	C12—C13	1.370 (3)
C15—C10	1.402 (2)	C12—H11	0.9300
C15—H14	0.9300	C14—O3	1.366 (2)
C10—C11	1.392 (2)	C14—C13	1.395 (3)
C10—C9	1.476 (2)	C13—H12	0.9300
C11—O1	1.371 (2)	C7—O1	1.449 (2)
C11—C12	1.393 (2)	C7—C8	1.515 (2)
C5—C6	1.381 (3)	C7—H7	0.9800
C5—C4	1.380 (3)	C9—O2	1.219 (2)
C5—C7	1.503 (2)	C9—C8	1.502 (2)
C4—C3	1.383 (3)	C8—H8B	0.9700
C4—H4	0.9300	C8—H8A	0.9700
C3—C2	1.367 (4)	O3—C16	1.418 (3)
C3—H3	0.9300	C16—H15A	0.9600
C2—C1	1.373 (4)	C16—H15B	0.9600
C6—C1	1.383 (3)	C16—H15C	0.9600
C6—H6	0.9300		
			
C14—C15—C10	120.12 (17)	C15—C14—O3	125.69 (19)
C14—C15—H14	119.9	C15—C14—C13	119.31 (17)
C10—C15—H14	119.9	O3—C14—C13	115.00 (18)
C11—C10—C15	119.75 (16)	C12—C13—C14	121.37 (17)
C11—C10—C9	119.71 (15)	C12—C13—H12	119.3
C15—C10—C9	120.51 (15)	C14—C13—H12	119.3
O1—C11—C10	122.90 (15)	O1—C7—C5	108.13 (13)
O1—C11—C12	117.23 (15)	O1—C7—C8	109.43 (14)
C10—C11—C12	119.86 (16)	C5—C7—C8	112.84 (14)
C6—C5—C4	118.74 (19)	O1—C7—H7	108.8
C6—C5—C7	119.48 (18)	C5—C7—H7	108.8
C4—C5—C7	121.74 (16)	C8—C7—H7	108.8
C5—C4—C3	120.9 (2)	O2—C9—C10	122.50 (16)
C5—C4—H4	119.5	O2—C9—C8	122.51 (16)
C3—C4—H4	119.5	C10—C9—C8	114.97 (14)
C2—C3—C4	119.2 (2)	C7—C8—C9	111.46 (14)
C2—C3—H3	120.4	C7—C8—H8B	109.3
C4—C3—H3	120.4	C9—C8—H8B	109.3
C3—C2—C1	121.2 (2)	C7—C8—H8A	109.3
C3—C2—Cl1	119.3 (2)	C9—C8—H8A	109.3
C1—C2—Cl1	119.45 (19)	H8B—C8—H8A	108.0
C5—C6—C1	120.8 (2)	C11—O1—C7	112.96 (12)
C5—C6—H6	119.6	C14—O3—C16	117.73 (18)
C1—C6—H6	119.6	O3—C16—H15A	109.5
C2—C1—C6	119.1 (2)	O3—C16—H15B	109.5
C2—C1—H1	120.4	H15A—C16—H15B	109.5
C6—C1—H1	120.4	O3—C16—H15C	109.5
C13—C12—C11	119.55 (18)	H15A—C16—H15C	109.5
C13—C12—H11	120.2	H15B—C16—H15C	109.5
C11—C12—H11	120.2		
			
C14—C15—C10—C11	0.1 (3)	C15—C14—C13—C12	−1.5 (3)
C14—C15—C10—C9	−178.06 (16)	O3—C14—C13—C12	178.56 (19)
C15—C10—C11—O1	177.30 (15)	C6—C5—C7—O1	142.77 (18)
C9—C10—C11—O1	−4.6 (2)	C4—C5—C7—O1	−39.4 (2)
C15—C10—C11—C12	−1.9 (3)	C6—C5—C7—C8	−96.0 (2)
C9—C10—C11—C12	176.21 (15)	C4—C5—C7—C8	81.8 (2)
C6—C5—C4—C3	−1.1 (3)	C11—C10—C9—O2	−175.33 (17)
C7—C5—C4—C3	−178.99 (18)	C15—C10—C9—O2	2.8 (3)
C5—C4—C3—C2	0.6 (3)	C11—C10—C9—C8	3.1 (2)
C4—C3—C2—C1	0.7 (4)	C15—C10—C9—C8	−178.80 (15)
C4—C3—C2—Cl1	−178.13 (16)	O1—C7—C8—C9	−57.54 (18)
C4—C5—C6—C1	0.4 (3)	C5—C7—C8—C9	−177.98 (14)
C7—C5—C6—C1	178.3 (2)	O2—C9—C8—C7	−154.04 (17)
C3—C2—C1—C6	−1.4 (4)	C10—C9—C8—C7	27.6 (2)
Cl1—C2—C1—C6	177.40 (19)	C10—C11—O1—C7	−26.7 (2)
C5—C6—C1—C2	0.9 (4)	C12—C11—O1—C7	152.50 (16)
O1—C11—C12—C13	−177.23 (17)	C5—C7—O1—C11	−179.56 (13)
C10—C11—C12—C13	2.0 (3)	C8—C7—O1—C11	57.17 (18)
C10—C15—C14—O3	−178.47 (18)	C15—C14—O3—C16	3.6 (3)
C10—C15—C14—C13	1.6 (3)	C13—C14—O3—C16	−176.6 (2)
C11—C12—C13—C14	−0.3 (3)		

### Hydrogen-bond geometry (Å, °)

**Table:**

*Cg*1 is the centroid of the C7–C12 ring.

**Table:**

*D*—H···*A*	*D*—H	H···*A*	*D*···*A*	*D*—H···*A*
C7—H7···O2^i^	0.98	2.50	3.260 (2)	135
C8—H8B···Cg1^ii^	0.97	2.69	3.5709 (18)	151

Symmetry codes: (i) −*x*+1, −*y*+1, −*z*+2; (ii) *x*+1, *y*, *z*.

## References

[bb1] Allen, F. H., Kennard, O., Watson, D. G., Brammer, L., Orpen, A. G. & Taylor, R. (1987). *J. Chem. Soc. Perkin Trans. 2*, pp. S1–19.

[bb2] Brito, I., Bórquez, J., Loyola, L. A. & López-Rodríguez, M. (2008). *Acta Cryst.* E**64**, o285. 10.1107/S1600536807066494 PMC2915337 21200851

[bb3] Brooks, G. T. (1998). *Pestic. Sci.* **22**, 41–50.

[bb4] Butcher, R. J., Jasinski, J. P., Yathirajan, H. S., Narayana, B. & Samshad (2007). *Acta Cryst.* E**63**, o3412–o3413.

[bb5] Chenera, B., West, M. L., Finkelstein, J. A. & Dreyer, G. B. J. (1993). *J. Org. Chem.* **58**, 5605–5606.

[bb6] Ellis, G. P. (1997). *Chromenes, Chromanones and Chromones.* New York: John Wiley and Sons Inc.

[bb7] Gabor, M. (1988). *The Pharmacology of Benzopyrone Derivatives and Related Compounds*, pp. 91–126. Budapest: Akademiai Kiado.

[bb8] Hao, L., Chen, J. & Zhang, X. (2010). *Acta Cryst.* E**66**, o1564. 10.1107/S1600536810020453 PMC3007079 21587807

[bb9] Hatakeyama, S., Ochi, N., Numata, H. & Takano, S. (1988). *J. Chem. Soc. Chem. Commun.* pp. 1022–1024.

[bb10] Hyana, T. & Saimoto, H. (1987). Jpn Patent JP 621 812 768.

[bb11] Kooijman, H., Spek, A. L., Kleijn, H., van Maanen, H. L., Jastrzebski, J. T. B. H. & van Kozikowski, A. P. (1984). *Acc. Chem. Res.* **17**, 410–416.

[bb12] Li, H.-Q., Xiao, Z.-P., Han, Y., Fang, R.-Q. & Zhu, H.-L. (2007). *Acta Cryst.* E**63**, o3923.

[bb13] Liu, C.-B., Chen, Y.-H., Zhou, X.-Y., Ding, L. & Wen, H.-L. (2007). *Acta Cryst.* E**63**, o90–o91.

[bb14] Nallasivam, A., Nethaji, M., Vembu, N., Ragunathan, V. & Sulochana, N. (2009). *Acta Cryst.* E**65**, o504–o505. 10.1107/S1600536809003948 PMC2968465 21582168

[bb15] Oxford Diffraction (2007). *CrysAlis PRO* and *CrysAlis RED* Oxford Diffraction Ltd, Abingdon, England.

[bb16] Sheldrick, G. M. (2008). *Acta Cryst.* A**64**, 112–122. 10.1107/S0108767307043930 18156677

[bb17] Spek, A. L. (2009). *Acta Cryst.* D**65**, 148–155. 10.1107/S090744490804362X PMC2631630 19171970

[bb18] Tang, Q.-G., Wu, W.-Y., He, W., Sun, H.-S. & Guo, C. (2007). *Acta Cryst.* E**63**, o1437–o1438.

[bb19] Valenti, P., Da Re, P., Rampa, A., Montanari, P., Carrara, M. & Cima, L. (1993). *Anticancer Drug. Des.* **8**, 349–360. 8251042

